# A retrospective cohort study on the bidirectional association between depression and chronic kidney disease

**DOI:** 10.3389/fneph.2025.1743594

**Published:** 2026-01-05

**Authors:** Ki Jin Jeun, Todd Brothers, Khaled Shawwa, Mohammad A. Al-Mamun

**Affiliations:** 1 Department of Pharmaceutical Systems and Policy, West Virginia University School of Pharmacy, Morgantown, WV, United States; 2 Pharmacy Practice and Clinical Research, College of Pharmacy, University of Rhode Island, Kingston, RI, United States; 3 Division of Nephrology, Department of Medicine, West Virginia University School of Medicine, Morgantown, WV, United States

**Keywords:** anxiety, chronic kidney disease, depression, disease progression, electronic health records

## Abstract

**Rationale and objectives:**

Depression has been associated with worse clinical outcomes in individuals with chronic kidney disease (CKD), yet its influence on kidney disease progression in earlier CKD stages remains underexplored. Thus, this study investigates the role of depression on CKD progression by stages, and bidirectional relationship using real-world data.

**Methods:**

This was a retrospective cohort analysis. Data was extracted from the TriNetX EMR database from 2007 to 2022. Patients (>18 years of age) with diagnosis of CKD were selected for the study. Key independent variables were diagnosis of depression or anxiety, identified by ICD codes, for the primary objective, and CKD stages (i.e., >3, 4, and 5) defined by KDIGO for the secondary objective. Primary outcome was progression to kidney disease (eGFR < 60 ml/min/bsa and > 40% decline in eGFR from the initial screening), and the secondary outcome was diagnosis of depression. Kaplan-Meier analysis and Cox proportional hazards model were used to evaluate the relationship between the dependent and independent variables while adjusting for covariates (sex, race, ethnicity, and age).

**Results:**

Depression was significantly associated with a higher risk of kidney disease progression (HR = 1.94 [1.77–2.11], p<0.001). Among patients with CKD, patients with CKD stages 4 and 5 had significantly higher risks (HR = 1.26 [1.17–1.35] and 1.38 [1.23–1.54], p<0.001) of new diagnosis of depression than those in stage ≤3, respectively. These associations remained statistically significant after matching and adjusting for age, sex, race, and comorbidities.

**Conclusion:**

Depression significantly accelerates CKD progression and patients with stage 5 CKD had the highest risk of developing depression. Our study advocates for integrating frequent mental health screenings for patients with CKD. This could improve patient outcomes and minimize negative consequences associated with depression.

## 1 Introduction

Chronic Kidney Disease (CKD) poses a substantial global health burden, with an estimated prevalence of over 800 million individuals worldwide and over 35 million in the United States (1–7). Beyond its clinical and economic burden, CKD is increasingly recognized as a condition influenced by complex interactions between physical and psychological health (8–13).

Among psychiatric conditions, depression is notably prevalent, with rates ranging from 20-40% in CKD populations which are substantially higher than in the general population (14–18). Anxiety, while also common and frequently co-morbid with depression, represents a distinct construct with different symptom patterns and clinical trajectories (17, 19). To maintain conceptual clarity, the present study focuses primarily on depression while acknowledging its frequent co-occurrence with anxiety in CKD.

Although depression is well characterized in advanced CKD and end-stage kidney disease (ESKD), its role in earlier CKD stages remains insufficiently understood despite the potential for meaningful intervention during this period (20–25). Recent work, including large-scale observational studies and KDIGO 2024 guidance, emphasizes the importance of early identification and management of psychosocial factors that may accelerate disease progression (25–27). Early-stage CKD (stages 1–3) represents a critical window in which modifiable behavioral and biological contributors may shape long-term outcomes, yet epidemiologic and mechanistic evidence in this population is limited.

Multiple pathways may plausibly link depression to CKD progression. Inflammation is a key mechanism: depression is associated with elevated pro-inflammatory cytokines such as IL-6 and TNF-α, which may contribute to renal microvascular injury and faster decline in estimated glomerular filtration rate (eGFR) (17, 19, 28). Neuroendocrine dysregulation, including chronic activation of the hypothalamic–pituitary–adrenal (HPA) axis and heightened sympathetic drive, can worsen hypertension, metabolic dysfunction, and endothelial injury which are all known accelerators of CKD progression. Additional behavioral mechanisms include reduced medication adherence, decreased engagement in self-management behaviors, suboptimal dietary adherence, and delayed follow-up, all of which may amplify renal risk (17, 19, 28). These mechanistic pathways collectively provide a strong theoretical basis for evaluating depression as a potentially modifiable risk factor for CKD progression.

Importantly, accumulating evidence across psychiatric research reinforces the interplay between mental health and physical disease. For example, real-world outpatient studies have illustrated the bidirectional relationships between psychiatric illness and metabolic comorbidities, highlighting the need for integrated care models (29). Likewise, emotional dysregulation, as demonstrated in studies examining guilt and affective symptoms in eating disorders, can exert broad effects on health-related behaviors and outcomes (30). The complexity of psychiatric diagnoses in real-world settings, including diagnostic variability and ethical considerations, further underscores the need for high-quality longitudinal data when evaluating depression’s impact on chronic conditions such as CKD (30). These insights parallel the challenges and opportunities inherent in using electronic health record (EHR) data, as seen in other retrospective real-world studies (8, 9).

Given these biological, behavioral, and clinical considerations, understanding the influence of depression during the earlier stages of CKD may have substantial implications for prevention, risk stratification, and timely mental health intervention. However, existing research remains limited, particularly regarding the temporal relationship between incident depression and subsequent kidney function decline. To address these gaps, we conducted a real-world, retrospective cohort study using electronic health record data to evaluate (1) whether patients diagnosed with depression after CKD experience accelerated kidney function decline, and (2) whether more advanced CKD stages are associated with a higher likelihood of developing new-onset depression.

## 2 Methods

This study was a retrospective cohort analysis utilizing data from the TriNetX electronic medical record (EMR) database, covering the years 2007 to 2022.

### 2.1 Data source

The data for this study were obtained from the TriNetX Research Network, a global repository of de-identified electronic medical records (EMR) data from various healthcare organizations (HCOs). This platform provides comprehensive information on demographics, diagnoses, procedures, laboratory results, and medications. Our analysis focused on data provided by West Virginia University, encompassing 2,222,520 patient records. As this study utilized fully de- identified patient data, it was not considered a human subject research, thus, did not require an Institutional Review Board (IRB) approval. Specifically, we utilized data from the diagnosis table (to track dates and CKD diagnosis), medication table (to identify antidepressant use and start dates), lab results (to monitor CKD progression via estimated glomerular filtration rate (eGFR) values), encounter and procedure files (to determine the last date of record), and patient demographics files. These datasets were integrated using de-identified patient IDs to compile the necessary information.

### 2.2 Study sample

A total of 90,602 CKD patients (aged >18 years) were identified using International Classification of Diseases (ICD) codes. The Ninth Edition (ICD-9-CM, code 581) was applied for data prior to 2016, and the Tenth Edition (ICD-10-CM, code N18) was used for data spanning from 2017 to 2022. We included adult patients (>18 years) with baseline CKD and at least one year of follow-up data. In this study, CKD staging was determined using eGFR values, based on two consecutive measurements recorded between 91 and 730 days apart, and categorized according to the 2024 KDIGO (Kidney Disease Improving Global Outcomes) guidelines (25–27). Of the two consecutive measurements, the date of the second measurement was taken as the index date, when the stage and kidney disease progression can be confirmed. eGFR values greater than 180 ml/min/body surface area (bsa) were excluded. Patients without eGFR data or whose CKD stage could not be determined were excluded from the cohort. Additionally, patients with only eGFR >90 (stage 1) were excluded, as their eGFR values could not be differentiated from those of individuals without CKD.

Several exclusion criteria were applied to minimize confounding biases related to depression and/or CKD progression. Firstly, patients with less than 1 year of follow-up data were excluded. Secondly, patients were excluded if they had a diagnosis of one of the top five most common cancers either prior to or within 10 years following their initial CKD diagnosis (31–34). Similarly, patients were excluded if they had a diagnosis of one of the five most prevalent mental illnesses within 3 years before or 10 years after their CKD diagnosis. Cancer and mental illness diagnoses were identified using ICD-10-CM and ICD-9-CM codes, which included: breast cancer (C50; 174), lung cancer (C34; 162), colon cancer (C18-20; 153-154), kidney cancer (C64-66; 189), prostate cancer (C61; 185), dementia (F03; 290), affective disorder (F39; 296.9), adjustment disorder (F43; 309), sleep disorder (F51; 307.4), and bipolar disorder (F31; 296.0, 296.4-7).

**Figure 1:**
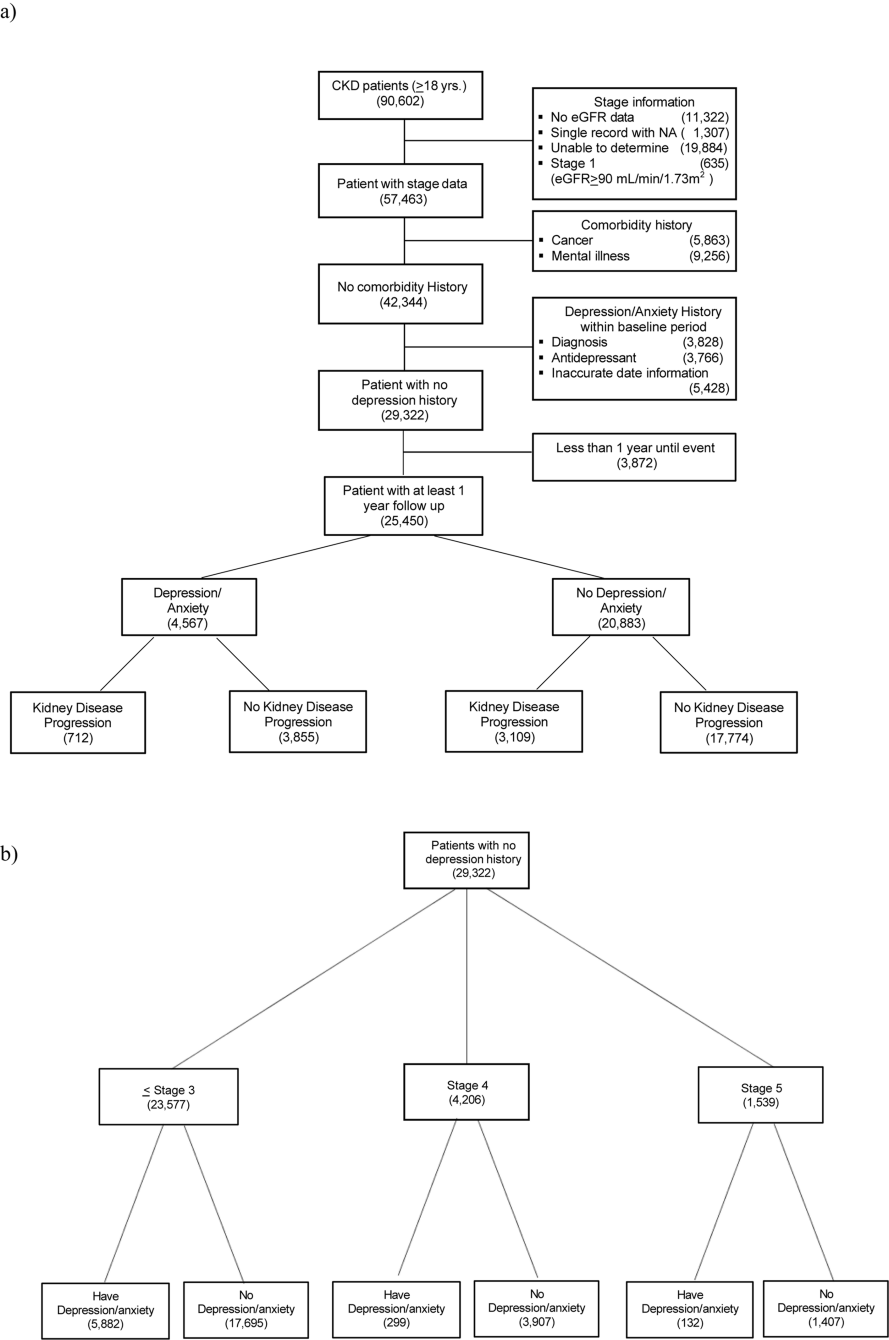
Flow diagram of study design. **(A)** Flow diagram of study samples for primary objective. Baseline period: before 1 year or within 90 days after CKD diagnosis. Follow-up period: 10 years. Cancer patients: breast cancer, prostate cancer, colon cancer, kidney cancer, lung cancer Mental comorbidity) dementia, adjustment disorder, affective disorder, bipolar disorder, sleep disorder. **(B)** Flow diagram of study samples for secondary objective.

To further reduce bias, patients with a history of depression or antidepressant prescription use during the baseline period (defined as before 1 year or within 90 days after CKD diagnosis) were excluded. This process is illustrated in **Figure 1**. The cohort was categorized separately for each objective. The primary objective cohort was dichotomized into depression or no depression (**Figure 1A**). The secondary objective cohort was categorized by CKD stages (e.g., ¾3, 4, and 5) (**Figure 1B**).

### 2.3 Definition of variables

The term ‘depression group’ refers to patients with a diagnosis of depression, anxiety, or both. Depression and anxiety were identified using ICD-9-CM codes (296 and 300) and ICD-10-CM codes (F32, F33, F40, and F41). We chose this approach because depression and anxiety frequently co-occur in CKD populations and share overlapping symptom pathways, treatment patterns, and behavioral consequences that may influence kidney health (17, 19). Patients were followed from the index date (i.e., initial CKD diagnosis) until they experienced kidney disease progression. Kidney disease progression was defined as an eGFR <60 mL/min/1.73 m² accompanied by a >40% decline from the initial screened value (35, 36). This definition aligns with KDIGO-endorsed surrogate endpoints and has been widely adopted in observational and clinical trial research, as it captures clinically meaningful deterioration while reducing the likelihood that short-term biological fluctuations or transient acute changes are misclassified as true progression.

To further mitigate measurement variability, progression required two eGFR values obtained at least 91 days apart, ensuring that acute illness or laboratory variability were less likely to influence outcome classification. We acknowledge, however, that EHR-derived laboratory measurements are subject to variable timing and frequency, reflecting real-world practice rather than standardized study protocols. As such, some imprecision in determining the exact onset of progression is unavoidable and may introduce interval censoring; this limitation should be considered when interpreting our findings.

If patients did not meet the above criteria, they were classified as having no kidney disease progression. In the depression cohort, time to kidney disease progression was calculated as the number of days between the initial depression diagnosis date and the event date. In the no-depression cohort, time to kidney disease progression was calculated as the number of days between the initial CKD diagnosis date and the event date. Patients were censored at the date of their last medical record entry (e.g., encounter, laboratory, procedure, or diagnosis record) or date of death.

For the secondary objective, the study cohort was divided into three groups based on CKD stages: Stage ≤3, Stage 4, and Stage 5, as defined by the KDIGO guidelines using eGFR values. These patients were followed up until the first depression event. For patients who developed depression, we identified the specific CKD stage at which depression onset occurred. The time to depression was calculated as the number of days from the initial date of the identified stage to the depression diagnosis date. For patients who did not develop depression, the number of days was calculated from the initial stage date to the next stage date. If no subsequent stage was recorded, the number of days were calculated using the last date of record.

### 2.4 Outcomes

The outcome variables were kidney disease progression for the primary objective and depression for the secondary objective.

### 2.5 Statistical analysis

Descriptive statistics were used to summarize the study cohort, including their demographic characteristics and study variables. Categorical variables were presented as frequencies and percentages, while continuous variables were presented as means. Baseline characteristics were compared using Pearson chi-square tests for categorical variables and independent-samples t- tests for continuous variables when comparing the depression and no-depression groups.

A propensity matching analysis was performed to balance the comparison groups on potential confounding variables, ensuring that the groups were comparable in key characteristics, thereby reducing bias in estimating the effect of depression on outcomes in the primary objective. A 1:1 propensity score model was developed using logistic regression, with the outcome variable being the presence of depression. Prior to conducting the propensity score matching, missing age data were imputed using the Multivariate Imputation by Chained Equations (MICE) method with Classification and Regression Trees (CART). The propensity score model included demographic variables (age, sex, race, and ethnicity) and three comorbidities (hypertension, hyperlipidemia, and type 2 diabetes). The distribution of the sample before and after matching is presented in Appendix A.

Kaplan-Meier analysis was performed to compare the time to event of the groups for both objectives. Additionally, a Cox proportional hazards model (CoxPH) was used to analyze the primary and secondary outcomes while adjusting for covariates (sex, race, ethnicity, and age). The proportional hazards assumption was assessed using Schoenfeld residuals for time-dependent covariates and none of the covariates were significant (p-value < 0.05). The Cox model was adjusted for the same covariates used in the propensity score matching. This adjustment was performed to account for any residual confounding that may persist following matching and to improve the precision of the estimated associations. The risks for kidney disease progression and depression were presented as Hazard Ratios (HR) with 95% confidence intervals. We have conducted a subgroup analysis to examine the types of antidepressants prescribed to CKD patients, comparing those who developed depression before versus after their CKD diagnosis. The list of drug names included for each category is available in Appendix B. All statistical analyses were conducted using R (version 4.2.2, R Foundation for Statistical Computing, Vienna, Austria). The main packages used in this analysis included: *dplyr, MatchIt, survival, and mice* (37–40).

## 3 Results

### 3.1 Baseline characteristics of patients

**Table 1** presents the baseline characteristics of the cohorts with and without depression. The table highlights significant differences in demographic and clinical characteristics between these groups. Notably, a higher percentage of females were in the depression group (61.6%) and mostly males were in the no-depression group (55.4%). Although the no-depression cohort initially exhibited a higher prevalence of all included comorbidities, after matching, only atrial fibrillation, anemia, and vitamin D deficiency remained significantly different. The differences in COPD, heart failure, nephrotic disease, and obesity became insignificant after matching.

**Table 1:** Baseline characteristics of the chronic kidney disease population: comparison between those with and without depression/anxiety.

	Before matching			After matching		
	Depression/ anxiety	No depression/ anxiety	P.overall	No depression/ anxiety	P.overall	SMD
	N=4567	N=20883		N=4567		
Sex			<0.001		0.95	0.002
Female	2811 (61.6%)	9303 (44.5%)		2807 (61.5%)		
Male	1756 (38.4%)	11576 (55.4%)		1760 (38.5%)		
Ethnicity			<0.001		0.90	0.009
Not Hispanic or Latino	4393 (96.2%)	18392 (88.1%)		4396 (96.3%)		
Hispanic or Latino	11 (0.24%)	88 (0.42%)		9 (0.20%)		
Unknown	163 (3.57%)	2403 (11.5%)		162 (3.55%)		
Race			<0.001		1.00	0.002
White	4319 (94.6%)	18523 (88.7%)		4321 (94.6%)		
Non-White	119 (2.61%)	786 (3.76%)		118 (2.58%)		
Unknown	129 (2.82%)	1574 (7.54%)		128 (2.80%)		
Age group			<0.001		0.81	0.001
18-44	101 (2.32%)	998 (2.44%)		93 (2.29%)		
45-64	894 (20.5%)	6047 (14.8%)		811 (20.0%)		
>65	3357 (77.1%)	33798 (82.8%)		3155 (77.7%)		
CKD Stage			<0.001		0.88	0.009
< Stage 3	4115 (90.1%)	15850 (75.9%)		4110 (89.99%)		
Stage 4	218 (4.78%)	2276 (10.9%)		216 (4.73%)		
Stage 5	235 (5.15%)	2757 (13.2%)		241 (5.28%)		
Kidney Disease Progression			0.24		0.80	
No	3855 (84.4%)	17774 (85.1%)		3845 (84.2%)		
Yes	712 (15.6%)	3109 (14.9%)		722 (15.8%)		
Comorbidities						
AFB:	313 (6.85%)	2336 (11.2%)	<0.001	388 (8.50%)	0.004	0.062
Anemia:	347 (7.60%)	2245 (10.8%)	<0.001	422 (9.24%)	0.005	0.059
COPD:	253 (5.54%)	1490 (7.13%)	<0.001	259 (5.67%)	0.820	0.006
HF:	353 (7.73%)	2351 (11.3%)	<0.001	394 (8.63%)	0.127	0.033
HLD:	1271 (27.8%)	7542 (36.1%)	<0.001	1273 (27.9%)	0.981	0.001
HTN:	1817 (39.8%)	10939 (52.4%)	<0.001	1818 (39.8%)	1.000	<0.001
ND:	752 (16.5%)	4346 (20.8%)	<0.001	754 (16.5%)	0.978	0.001
Obesity:	484 (10.6%)	2522 (12.1%)	<0.001	477 (10.4%)	0.838	0.005
T2DM:	1072 (23.5%)	6381 (30.6%)	<0.001	1066 (23.3%)	0.902	0.003
VDD:	313 (6.85%)	2336 (11.2%)	<0.001	388 (8.50%)	0.004	0.062

Matched for: age group, sex, race, ethnicity, HTN, HLD, and T2DM.

AFB, atrial fibrillation; COPD, chronic obstructive pulmonary disease; HF, heart faliure; HLD, hyperlipidemia; HTN, hypertension; ND, nicotine dependence; T2DM, type 2 diabetes mellitus; VDD, vitamin D deficiency.

### 3.2 Effect of depression on CKD progression

**Figure 2:**
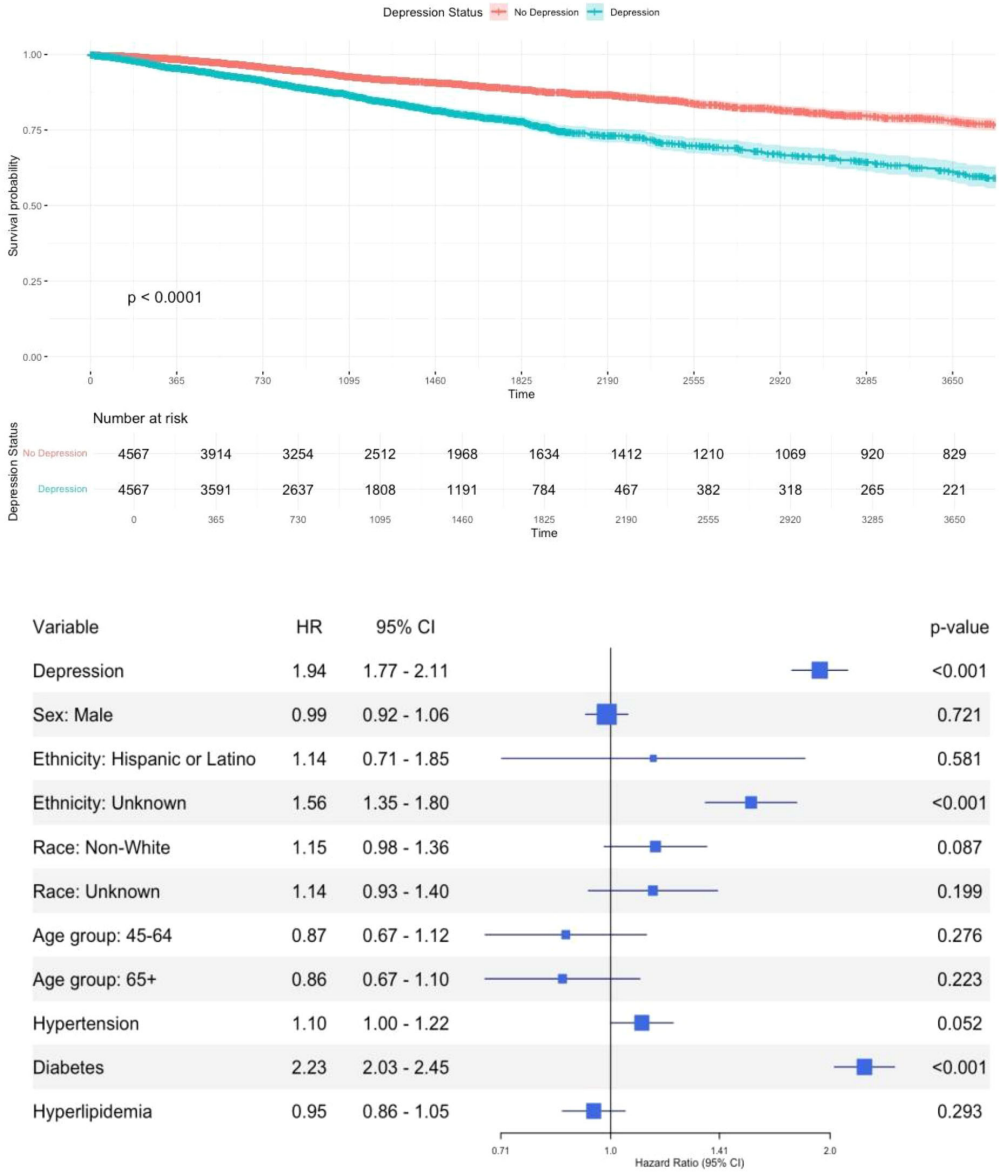
Time-to-event model visualization: time to kidney disease progression (depression vs. no depression). HR, hazardous ratio; CI, confidence interval; Non-White includes American Indian, Black/African American, Asian, and Pacific Islander individuals.

In the Kaplan–Meier curve, the probability represents the percentage of the population who had kidney disease progression over the observed time period. Patients with depression progressed to kidney disease faster compared to patients without (**Figure 2**). The difference between the survival curves is statistically significant (p<0.0001). This relationship is further illustrated in the adjusted Cox model. Depression was significantly associated with a higher risk of kidney disease progression 1.94 (95% CI = 1.77–2.11, p<0.05). Additionally, patients with diabetes have more than twice the risk of progressing to CRI compared to those without diabetes (HR = 2.23 [2.03–2.45], p<0.05).

### 3.3 Impact of CKD on the development of depression

**Figure 3:**
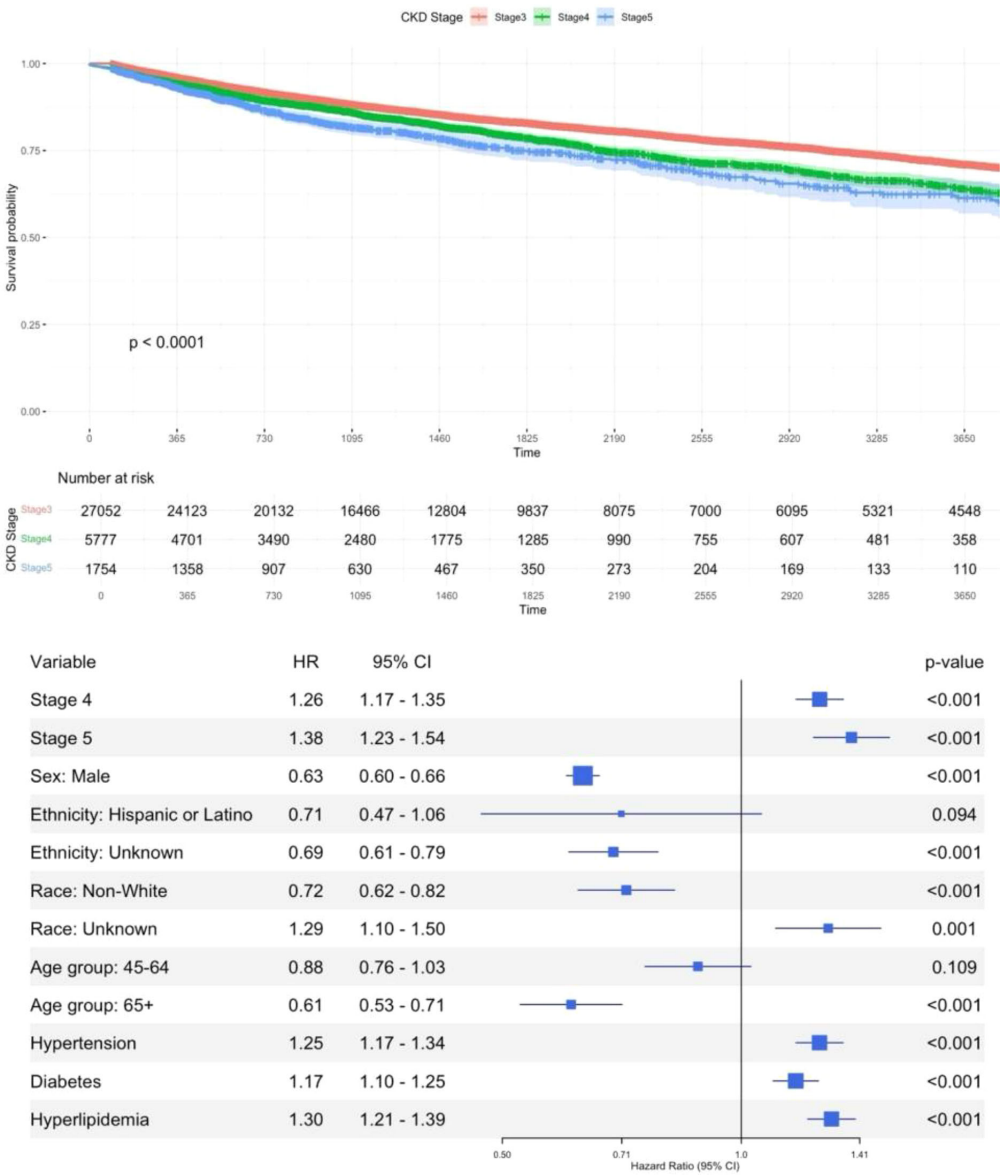
Time to event model visualization – time to depression by CKD stage groups. HR, hazardous ratio; CI, confidence interval; Non-White includes American Indian, Black/African American, Asian, and Pacific Islander individuals.

**Figure 3** illustrates the time to depression onset across different stages of CKD: Stage ≤3 (blue line), Stage 4 (green line), and Stage 5 (red line). Among the three CKD stage groups, the survival probability decreases most rapidly for patients in Stage 5, followed by Stage 4, and then Stage ≤3. This indicates that patients in more advanced stages of CKD are more likely to develop depression earlier. The differences between the survival curves for the CKD stages are statistically significant (p<0.0001), suggesting a strong association between CKD stage and the time to depression onset.

This relationship is further highlighted in the forest plot. Patients in Stage 4 had a higher risk of developing depression compared to those in Stage ≤3 (HR = 1.26 [1.17–1.35], p<0.05), and those in Stage 5 had an even higher risk (HR = 1.38 [1.23–1.54], p<0.05). Additionally, several factors were found to be associated with the risk of developing depression. Males were at a 39% lower risk of developing depression compared to females (CI = 0.60–0.66, p<0.05), and individuals of non-White race had a 28% lower risk compared to those of White race (CI = 0.68–0.77, p<0.05). The hazard ratios also indicated that comorbidities significantly increased the risk of depression: hypertension (HR = 1.25 [1.17–1.34], p<0.05), diabetes (HR = 1.17 [1.10–1.25], p<0.05), and hyperlipidemia (HR = 1.30 [1.21–1.39], p<0.05).

### 3.4 Utilization of antidepressants among CKD patients

**Figure 4:**
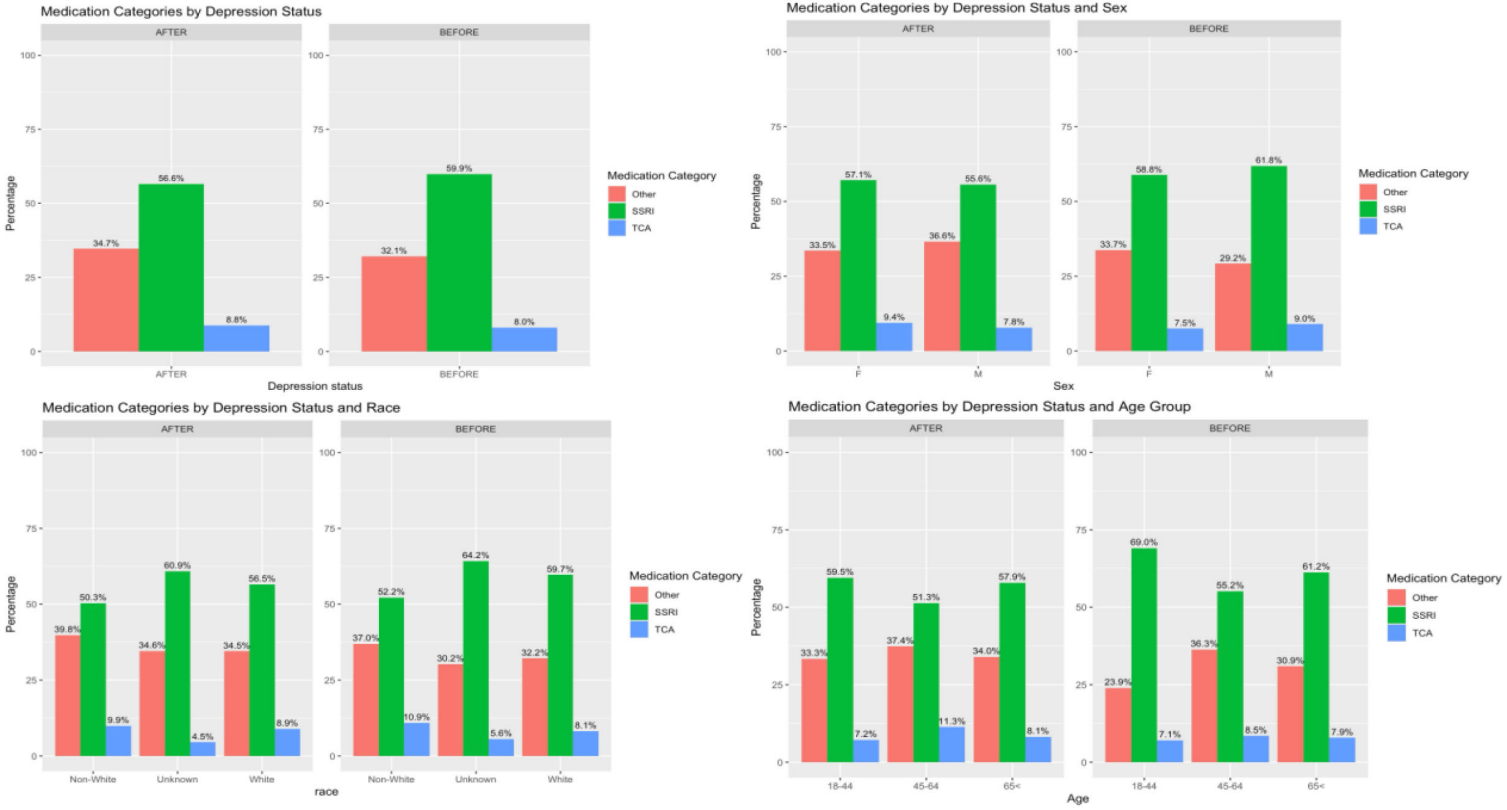
Types of antidepressants were prescribed before and after chronic kidney disease.

The comparison of types of antidepressants taken by patients who were diagnosed with depression before and after CKD is displayed in **Figure 4**. Selective serotonin reuptake inhibitors (SSRI) were the most prescribed antidepressant across all age, sex, and ethnicity. When comparing the composition among sex, there was higher percentage of SSRI prescribed among male, and female had a higher percentage in the other category among patients who were diagnosed with depression before CKD. This proportion changed among the patients who were diagnosed with depression after CKD, where more female was prescribed with SSRI, and more male was prescribed with other antidepressant. The percentage of SSRI prescriptions was lower among the patients who were diagnosed with depression after CKD across all age groups, and higher percentage was observed in both TCA and other antidepressant prescriptions.

## 4 Discussion

The findings of this study underscore a significant association between depression and CKD progression, particularly in the earlier stages. Our results show that patients with a diagnosis of depression following their CKD diagnosis had a higher likelihood of kidney disease progression compared to those without depression. Although no prior studies have specifically examined this relationship in earlier stages of CKD, existing literature has identified associations between depression and outcomes such as dialysis initiation and mortality. For example, a recent study by Shen et al. found that CKD patients with depressive symptoms were more likely to initiate dialysis or experience all-cause mortality, pointing to a potential link between mental health and disease trajectory (37). Similarly, Zhang et al. observed that depressive symptoms were associated with a 2.25-fold higher risk of progression to dialysis over a three-year period (38). These associations may be partly explained by reduced treatment adherence and behavioral changes commonly seen in individuals with depression (39).

Nonetheless, prior studies have produced conflicting findings (40, 41). Some concluded that depressive symptoms were not significantly related to disease progression or adverse outcomes like cardiovascular events (40, 42). While our hazard ratio analysis identified a statistically significant relationship between depression and progression of kidney disease, prior studies have reported varying magnitudes of association, possibly due to differences in study populations, access to mental health care, or measurement definitions (15). For instance, while we used eGFR decline and a threshold-based definition for kidney disease progression, other studies defined progression based on dialysis initiation or ESRD diagnosis. Population differences may also contribute: Eveleens et al., who focused on individuals aged 65 and older, reported contrasting findings. In our study, although the hazard ratio for CKD progression was numerically lower in those over 65, the difference was not statistically significant (41). Fischer et al. examined African American patients, whereas our cohort included individuals of multiple races, albeit predominantly White individuals, may explain some variation in our findings (40). Our analysis also identified a statistically significant association between CKD stage and subsequent depression diagnoses. Individuals in later CKD stages (Stages 4 and 5) were more likely to be diagnosed with depression than those in earlier stages. The Kaplan–Meier analysis indicated shorter time to depression onset in more advanced stages, suggesting a possible increasing psychological burden as kidney function declines. These results are consistent with prior studies reporting higher depression prevalence among individuals with advanced CKD. For example, Palmer et al.’s meta- analysis estimated depression prevalence at 22.8% to 39.3% in individuals with CKD, compared to lower rates in earlier stages (21.4% to 26.5%) (15).

Contrary findings have also been reported. For example, Balogun et al. found that depression was more prevalent among elderly male patients and in earlier CKD stages. They also reported that the prevalence of depression was higher in the earlier stages of CKD (29% to 47%) than in later stages (30%)^47^. However, these differences may be attributable to sample characteristics, such as Balogun’s use of a Veterans Affairs cohort composed primarily of older adults.

Additionally, their analysis focused on prevalence, while our study examined time-to-event and hazard ratios, limiting direct comparison.

From a clinical standpoint, implementing systematic depression screening could meaningfully change CKD care pathways. The patient health questionnaire (PHQ)-2 is a recommended first-line tool due to its brevity and sensitivity, with positive screens followed by the PHQ-9 to guide diagnostic confirmation and severity assessment (43, 44). For patients in CKD Stages 1–3, screening every 6–12 months may be appropriate, while those in Stages 4–5 may benefit from screening every 3–6 months due to higher psychological burden and increased risk of symptom progression. For individuals identified with clinically significant depressive symptoms, interventions such as collaborative care models, psychotherapy (e.g., CBT), antidepressant pharmacotherapy with renal-adjusted dosing, social work involvement, and structured adherence-support programs have demonstrated benefit in chronic medical conditions. Embedding mental health professionals within nephrology practices or using telehealth-supported behavioral health could further enhance access. These targeted strategies may help address the biological, behavioral, and socioeconomic pathways through which depression contributes to poorer kidney outcomes.

Strengths of our study include a large sample size, the use of propensity score matching and time-to-event modeling methods, an extended retrospective follow-up period, and inclusion of data from rural Appalachian populations, an often-underrepresented group. It is important to note that although depression was associated with accelerated CKD progression in our cohort, these findings do not establish causality. The observational nature of our study limits the ability to determine whether depression directly contributes to kidney function decline or whether unmeasured confounding, shared biological pathways, or the psychological burden of worsening CKD may explain part of this relationship. While inflammatory, behavioral, and socioeconomic mechanisms provide biologically plausible pathways, the observed associations should be interpreted as indicative rather than causal.

This study relied on ICD-9-CM and ICD-10-CM codes to identify depression, anxiety, and CKD, which is standard in large EHR-based analyses but introduces several important limitations. ICD-coded psychiatric diagnoses generally capture only clinically recognized and documented conditions and may underestimate milder, subclinical, or undiagnosed cases. Furthermore, because depression and anxiety frequently co-occur and cannot always be reliably distinguished using administrative codes, these conditions were combined into a single exposure variable. While this approach aligns with prior real-world database studies, it may obscure clinically meaningful differences in symptomatology and severity.

Similarly, CKD was identified using ICD codes and staged using eGFR values obtained from routine clinical practice, which are subject to variability in measurement timing, frequency, and clinical context. Although we excluded patients with insufficient laboratory data or physiologically implausible values, residual misclassification of CKD stage may still occur. Additionally, to reduce confounding, patients with major cancers, prevalent mental illnesses, or insufficient follow-up were excluded using ICD codes. While these decisions improved internal validity, they may limit generalizability and do not eliminate unmeasured confounding inherent in retrospective EHR research.

Several additional limitations merit discussion. First, the absence of socioeconomic variables limited our ability to adjust for key determinants that influence both mental health and CKD outcomes. Socioeconomic status affects access to primary and specialty care, medication affordability, health literacy, transportation, environmental exposures, and chronic psychosocial stress, all of which may shape the onset or progression of both depression and kidney disease. The lack of these variables may therefore have contributed to residual confounding and could partly explain some of the associations observed.

Second, our study population was predominantly White, which may limit generalizability to more racially and ethnically diverse populations who often experience different burdens of CKD, variation in mental health diagnosis and treatment patterns, and distinct structural or socioeconomic barriers.

Third, because the dataset was derived largely from inpatient encounters, outpatient psychiatric diagnoses, antidepressant prescriptions, and routine eGFR monitoring may have been under-ascertained. This may lead to misclassification of both exposure and outcome, particularly for individuals with milder or intermittently managed depression.

Fourth, although propensity score matching helped reduce observable selection bias, unmeasured confounding remains possible, especially in relation to socioeconomic factors that were unavailable in the dataset. As such, some of the observed associations may reflect structural or environmental disparities rather than direct clinical effects, and no causal inference can be made in a retrospective design.

Finally, our models relied on the documented timing of depression diagnoses and eGFR measurements within the EHR. In routine clinical practice, laboratory testing and diagnostic coding occur at irregular intervals, and depressive symptoms may precede formal documentation by weeks or months. This limits our ability to determine the precise temporal sequence between exposure and outcome, and the resulting interval censoring may influence hazard estimates. Future studies incorporating more granular socioeconomic data and analytic methods designed to account for irregular observation intervals may improve precision and interpretability.

Future research should also examine interventional strategies aimed at addressing the bidirectional relationship between depression and CKD. Approaches such as nephrology-adapted collaborative care models, behavioral activation or CBT-based programs, and adherence-support interventions may hold promise for improving both psychological well-being and renal outcomes. Pragmatic or randomized trials that integrate behavioral health services within CKD clinics, including telehealth-enabled models. are needed to determine whether treating depression can meaningfully enhance patient quality of life or potentially slow CKD progression.

In summary, while these limitations reflect the inherent constraints of real-world data and administrative coding practices, our study contributes to the growing body of literature demonstrating a significant association between depression and CKD progression and an increased likelihood of depression among individuals with more advanced CKD. Because this study was retrospective and observational, the temporal patterns identified should be interpreted as associations rather than causal effects. Although our findings suggest that depression is associated with a higher probability of CKD progression and that more advanced CKD stages are associated with a greater likelihood of depression, these relationships do not establish causation. Instead, they highlight clinically meaningful bidirectional associations that warrant further investigation through prospective or interventional studies. Collectively, these findings underscore the importance of incorporating routine mental health assessment into CKD care and support the need for targeted research aimed at improving both psychological and renal outcomes.

## Data Availability

The raw data supporting the conclusions of this article will be made available by the authors, without undue reservation.
